# Type 1 diabetes - an auto-inflammatory disease: a new concept, new therapeutical strategies

**DOI:** 10.1186/1479-5876-10-S3-I12

**Published:** 2012-11-28

**Authors:** Catarina Limbert

**Affiliations:** 1 Dept. of Pediatric Endocrinology and Diabetology, University Pediatric Hospital of Dona Estefania, Lisbon, Portugal

## 

Type 1 diabetes (T1D) is characterized by a progressive destruction of pancreatic beta-cells that results in absolute insulin deficiency and the need for daily insulin treatment. However, the auto-inflammatory nature of the disease has been recently demonstrated by understanding the precise cellular/molecular mechanisms of type 1 diabetes’ aetiology and progression. Studies in NOD mice have shown that the disease occurs as consequence of a breakdown in the immune-system balance. Although the detection of islet specific auto antibodies in *sera* from humans with T1D was the first sign of an autoimmune disease, there is strong evidence in both, mice and humans that auto-reactive T cells play a prominent role in disease onset and progression. Destruction of beta-cells is mainly due to the secretion of powerful pro-inflammatory cytokines, such as Interleukine -1 beta (IL-1b), tumor-necrosis-factor alpha (TNFα) and interferon gamma(IFNγ). Recent studies have shown that Foxp3 regulatory T cells’ (Treg) activity is diminished, which affects the fine balance of the peripheral self-tolerance and immune activation.

Another key concept is the fact that adult endocrine pancreas, like most tissues is dynamically regulated by expansion and reduction mechanisms. There is enough evidence for beta-cell regeneration in animal models. In humans, it is well-accepted that beta-cell regeneration occurs permanently during life and it depends on the current metabolic demand or pancreatic injury. At diabetes clinical onset, beta-cell dysfunction/destruction has overcome beta-cell regeneration and consequently hyperglycaemia occurs.

These findings suggest that immunomodulatory agents directed to the immune pathogenesis of T1D might enable preservation of insulin-secreting cells. Besides, induction of endogenous beta-cell regeneration and beta-cell replacement are promising strategies to attain insulin independence.

Nevertheless, specific immunological markers urge to be identified in order to assess the immunologic stage of the disease. These might enable therapeutic interventions targeting restoration of the immune balance, stimulation of beta-cell regeneration and thereby endogenous insulin secretion. Similar to neoplastic and HIV-associated diseases, the successful combination of drugs set a light in the tunnel leading to the cure of diabetes.

